# Collective behavior of oscillating electric dipoles

**DOI:** 10.1038/s41598-018-33990-y

**Published:** 2018-10-24

**Authors:** Simona Olmi, Matteo Gori, Irene Donato, Marco Pettini

**Affiliations:** 1 Inria Sophia Antipolis Méditerranée Research Centre, MathNeuro Team, 2004 route des Lucioles-Boíte Postale 93, 06902 Sophia Antipolis, Cedex France; 2 Institut für Theoretische Physik, Technische Universität Berlin, Hardenbergstr. 36, 10623 Berlin, Germany; 3 CNR - Consiglio Nazionale delle Ricerche – Istituto dei Sistemi Complessi, 50019 Sesto Fiorentino, Italy; 4 Aix Marseille Univ, CNRS, CPT, Marseille, France; 5 CNRS Centre de Physique Théorique UMR7332, 13288 Marseille, France

## Abstract

We investigate the dynamics of a population of identical biomolecules mimicked as electric dipoles with random orientations and positions in space and oscillating with their intrinsic frequencies. The biomolecules, beyond being coupled among themselves via the dipolar interaction, are also driven by a common external energy supply. A collective mode emerges by decreasing the average distance among the molecules as testified by the emergence of a clear peak in the power spectrum of the total dipole moment. This is due to a coherent vibration of the most part of the molecules at a frequency definitely larger than their own frequencies corresponding to a partial cluster synchronization of the biomolecules. These results can be verified experimentally via spectroscopic investigations of the strength of the intermolecular electrodynamic interactions, thus being able to test the possible biological relevance of the observed macroscopic mode.

## Introduction

The present work is motivated by a (practical) problem of (potentially) relevant impact: the experimental confirmation or refutation of the possibility of detecting long-range electrodynamic attractive forces among biomolecules, if any. The ultimate reason for searching these electrodynamic interactions stems from the observation of the high efficiency displayed by biomolecules when moving toward their specific targets and sites of action in living cells. Biochemical players “need to know” where to go and when, and are capable to reach their cognate partners so quickly that it hardly seems to be the result of a random search driven by thermal fluctuations (Brownian motion) alone. A longstanding hypothesis surmises that in order to accelerate these encounters, selective forces acting at a long distance are needed besides standard short-range ones (like covalent bonds, van der Waals forces etc.). This mechanism of molecular recruitment at a distance could be of high relevance to biology. Unfortunately, because of technological limitations, an experimental proof or refutation of this possibility has been for a long time and is still sorely lacking. The present day technological advances allow to cope with experimental challenges that were very hard to tackle in the past. This is the case of modern methods in Fluorescence Fluctuation Spectroscopy^1,2^ that we invoked in our previous studies^3–5^, and of Terahertz spectroscopy^6^ that we suggest for the present study as a reliable experimental setup to detect collective vibrating modes emerging in identical molecules ensembles.

In particular, in order to clarify the possible activation of electrodynamic interactions, it has been previously studied how the diffusion behavior of biomolecules in solution changes depending on their concentration, via the employment of Fluorescence Fluctuation Spectroscopy^3–5^. Varying the concentration corresponds to varying the average intermolecular distance as a consequence of the action of surmised electrodynamic intermolecular interactions. Moreover, is it shown in^7^ that, by shining a laser light on an aqueous solution of proteins a strong mesoscopic dipolar vibration for each molecule can be excited and detected in a sub-Terahertz frequency range. The detection in this case is possible thanks to dye molecules attached to each protein that harvest the incoming laser light. In particular these strong dipolar oscillations can switch-on intermolecular electrodynamic interactions possibly acting at a large distance (even up to some thousands of Angstroms).

In the following we put forward an alternative/complementary experimental strategy. To this aim, we study the dynamical behaviour emerging in an ensemble of identical molecules, each one vibrating with a low frequency mode, kept active by an external energy supply. A (mesoscopic) vibrational mode of a single molecule is the coherent vibration at the same frequency of a relevant fraction (or even of all) of its atoms, which brings about an oscillating electric dipole moment at the same frequency. The latter, in turn, activates an electrodynamic intermolecular force whose potential decays as −1/*r*^3^, where *r* is the intermolecular distance. Then, by changing the average distance among the molecules (as a control parameter), we have observed the emergence of a collective behaviour, this time involving the whole ensemble of molecules, which is detected through neat and substantial changes of a collective observable. This observable is a collective (macroscopic) one since it depends on the dynamical behaviour of all the molecules in the ensemble, and, more precisely, it consists in the power spectrum of the time variation of a quantity proportional to the total electric dipole moment of the system. In practice, this suggests a new experimental strategy to ascertain whether electrodynamic interactions among biomolecules can be activated: if we consider aqueous solutions of biomolecules, prepared with a salted solution sufficient to shield electrostatic interactions down to a few Angstroms, then we can excite these biomolecules with a suitable external forcing (like the laser light) in order to induce a mesoscopic vibration in the system at the level of the single molecule. Then, by changing the concentration of these solutions, that is by changing the intermolecular interaction strength which is a function of the distance between the molecules, and by switching on and off a laser light shining on the solution, some significative variation could be looked for by means of spectroscopic techniques to reveal whether or not the mentioned electrodynamic interactions are present.

The paper is organized as follows: in Section II the model is defined and discussed, while in Sec. III we report the outcomes of the Molecular Dynamics simulations of the model and we comment on the observed phenomenology. Section IV is devoted to some concluding remarks about the results presented throughout the present paper.

## The Model

### Model for the biomolecule

As already stated in the Introduction, the present work aims at understanding whether through spectroscopic experiments, presumably in the Terahertz frequency domain, an experimental confirmation or refutation can be obtained of the theoretical prediction firstly stated in^7^: whether or not it is possible to activate electrodynamic forces between biomolecules, vibrating out-of-thermal equilibrium, in aqueous solution. In what follows, we consider a simple model for an ensemble of biomolecules randomly oriented and randomly distributed in 3D space, coupled through an interaction potential decreasing as −1/*r*^3^ as a function of the interparticle distance *r*. Each biomolecule is modeled as an oscillating electric dipole composed of two material points, each of them with a mass *m* and the same absolute value *Ze* of the electric charge but with opposite sign. The positions of the positive and negative charged particles of the i-th biomolecule are respectively **r**_+,*i*_ and **r**_−,*i*_. The position of the center of mass of the *i*-th biomolecule is indicated by **R**_*i*_ while the direction of each dipole is

1 $${\hat{{\bf{r}}}}_{i}=\frac{{{\bf{r}}}_{+,i}-{{\bf{r}}}_{-,i}}{\Vert {{\bf{r}}}_{+,i}-{{\bf{r}}}_{-,i}\Vert }.$$

Both have been considered to be fixed, so that the charged particle of each biomolecule are constrained to oscillate along their joining line, i.e. d**R**_*i*_/d*t* = 0 and ${\rm{d}}{\hat{{\bf{r}}}}_{i}/dt=0$. These assumptions may seem to be quite strong with respect to a real biological molecular system where particles both diffuse and rotate due to the collisions with the surrounding water molecules. Indeed these assumptions are justified by the fact that the characteristic time scales of the inner oscillation of biomolecules are much shorter compared with those associated with the diffusion of the biomolecules centers of mass at their rotational diffusion (See Supplementary Information, Sec. I, for a more detailed discussion). It follows that the only dynamical variables are the mutual distances ${r}_{i}=({{\bf{r}}}_{+,i}-{{\bf{r}}}_{-,i})\cdot {\hat{{\bf{r}}}}_{i}$ between the two centers of charge of each biomolecule. The electric dipole moment is given by ${{\bf{p}}}_{i}(t)=Ze{r}_{i}(t){\hat{{\bf{r}}}}_{i}$.

Despite its simplicity, this model is suited to explore the presence of collective effects on the dynamics of coupled oscillating dipoles with fixed distance and orientations representing a system of oscillating biomolecules in mutual interaction through long-range quasi-static electrodynamic field generated by their oscillatory electric dipole.

#### Mechanical properties of a biomolecule

The mechanical properties of each biomolecule are described by an effective potential $V({r}_{i})={V}_{eff}(\Vert {{\bf{r}}}_{+,i}-{{\bf{r}}}_{-,i}\Vert )$ that is supposed to exist between material charged points. A minimum of the effective potential is assumed to be attained for *r*_*i*_ = *r*_*i*0_, i.e. d*V*/d*r*_*i*_(*r*_*i*0_) = 0 and ${{\rm{d}}}^{2}V\,{/\,\mathrm{dr}}_{i}^{2}({r}_{i0}) < 0$, so that the effective potential is assumed to be

2 $${V}_{eff}({r}_{i})\approx \frac{1}{2}m{\omega }_{i}^{2}{({r}_{i}-{r}_{i0})}^{2}+\frac{1}{4}m\frac{{\omega }_{i}^{2}}{{{\rm{\Lambda }}}^{2}}{({r}_{i}-{r}_{i0})}^{4},$$

where the parameter Λ is the characteristic length of the oscillation amplitude for the emergence of non-harmonic contributions. The non-harmonic contribution has been included for two main reasons: firstly, it accounts for the exchange of energy of the main collective mode with other vibrational normal modes of the biomolecule; secondly, it prevents instability of the oscillations when the electric dipoles are strongly coupled among them.

### Mutual quasi-electrostatic interactions among biomolecules

The physical system that we are modeling is an ensemble of oscillating biomolecules in aqueous solutions in presence of freely moving ions. Since this work aims at studying collective phenomena originated by long-range electrodynamic interactions among biomolecules, we neglect any electrostatic interaction. This assumption is well justified in presence of Debye screening which, inside living cells, has a length scale of a few Angstroms. It follows that, for the range of average intermolecular distances which is of interest here (that is, $\sim {10}^{2}\,\mbox{--}\,{10}^{3}{\rm{\AA }}$), the contribution of electrostatic fields is negligible. To the contrary, electrodynamic fields of sufficiently high frequency are not screened in water also in presence of freely moving ions, as it follows both from theory and dielectric spectroscopic experiments for *ω* > 2.5 × 10^2^ MHz^8^. As mentioned before the expected frequency for the collective oscillation of a biomolecule is around 0.1–1 THz, thus largely above the upper frequency threshold for important screening effects on electrodynamic fields. Collective phenomena are more probably expected in systems of resonant oscillators: for such a reason, a system of *N* identical biomolecules (oscillators) has been considered. Moreover, resonance of electric dipole oscillators, describing biomolecules, has been argued to be a necessary condition in order to activate long range dipole-dipole ($\sim {R}_{ij}^{-3}$) electrodynamic interactions^5^.

In our very simple model the force acting on each charge barycentre of the *i*-th electric dipole due to the *j*-th dipole is given by

3 $${{\bf{F}}}_{CED}({{\bf{r}}}_{\pm ,i};{{\bf{R}}}_{j})=Ze{{\bf{E}}}_{CED}({{\bf{r}}}_{\pm ,i};{{\bf{R}}}_{j})\,\mathrm{.}$$

where **E**_*CED*_(**r**; **R**_*j*_) is the value of the electric field in **r** generated by the *j*-th dipole whose center is in **R**_*j*_. According to the Classical Electrodynamics (CED), if we assume valid the dipole approximation, i.e. $\Vert {\bf{r}}-{{\bf{R}}}_{j}\Vert \gg {r}_{j}$, the expression for the electric field takes the form

4 $$\begin{array}{ccc}{{\bf{E}}}_{CED}({\bf{r}};{{\bf{R}}}_{j}) & = & {\int }_{0}^{+{\rm{\infty }}}\,{\rm{d}}\omega \,{\textstyle \tfrac{\exp [i\omega (t\pm \sqrt{\epsilon (\omega )}\parallel {\bf{r}}-{{\bf{R}}}_{j}\parallel /c)]}{4\pi \epsilon (\omega ){\parallel {\bf{r}}-{{\bf{R}}}_{j}\parallel }^{3}}}\\  &  & \times \,\{[3{\hat{{\bf{n}}}}_{j}({\bf{r}})({{\bf{p}}}_{j}(\omega )\cdot {\hat{{\bf{n}}}}_{j}({\bf{r}}))-{{\bf{p}}}_{j}(\omega )](1\mp {\textstyle \tfrac{i\omega \sqrt{\epsilon (\omega )}\parallel {\bf{r}}-{{\bf{R}}}_{j}\parallel }{c}})+\\  &  & -[{{\bf{p}}}_{j}(\omega )-{\hat{{\bf{n}}}}_{j}({\bf{r}})({{\bf{p}}}_{j}(\omega )\cdot {\hat{{\bf{n}}}}_{j}({\bf{r}}))]{\textstyle \tfrac{{\omega }^{2}\epsilon (\omega )\parallel {\bf{r}}-{{\bf{R}}}_{j}{\parallel }^{2}}{{c}^{2}}}\}.\end{array}$$

where *c* is the speed of light, ${\widehat{{\bf{n}}}}_{j}={\bf{r}}-{{\bf{R}}}_{j}/(\Vert {\bf{r}}-{{\bf{R}}}_{j}\Vert )$ is the direction joining the center of dipole **R**_*j*_ to *r*, **p**_*j*_(*ω*) is the Fourier Transform of the electric dipole moment of the *j*-th biomolecule in time domain and *ε*(*ω*) is the dielectric constant of the medium.

For the range of frequencies we explore ($\omega  \sim {\rm{\Omega }}\approx 1$ THz), the dielectric constant of an electrolytic aqueous solution can be assumed to be real $\Re e\,(\epsilon (\omega ))\gg \Im {\rm{m}}(\epsilon (\omega ))$ and approximatively constant *ε*_*WS*_(Ω) ≈ 3. Moreover both the intermolecular average distance *R*_*ij*_ ≈ 10^3^ Å and the characteristic linear dimensions *r*_0_ ≈ 10 Å are much smaller than the characteristic wavelength of the electromagnetic field $\lambda =2\pi c/(\epsilon \omega )\simeq 5\times {10}^{7}\,{\rm{\AA }}$. This allows to assume that the electromagnetic field has the same value for both centers of charge of each biomolecule, i.e. **E**_*CED*_(**r**_+,*i*_; **R**_*j*_) = **E**_*CED*_(**r**_−,*i*_, **R**_*j*_) = **E**_*CED*_(**R**_*i*_; **R**_*j*_), and that any retardation effect can be neglected, i.e. ${R}_{ij}/\lambda \ll 1$. With these approximations the acceleration of the *i*-th dipole is directed along ${\widehat{{\bf{r}}}}_{i}$ and due to the interaction with the *j*-th dipole reads as

5 $$\begin{array}{rcl}{(m\tfrac{{{\rm{d}}}^{2}{r}_{i}}{{\rm{d}}{t}^{2}})}_{CED} & = & {(m\tfrac{{{\rm{d}}}^{2}{{\bf{r}}}_{+,i}}{{\rm{d}}{t}^{2}}-m\tfrac{{{\rm{d}}}^{2}{{\bf{r}}}_{-,i}}{{\rm{d}}{t}^{2}})}_{CED}\cdot {\widehat{{\bf{r}}}}_{i}=2Ze\,\sum _{j\ne i}\,{{\bf{E}}}_{CED}({{\bf{R}}}_{i};{{\bf{R}}}_{j})\cdot {\widehat{{\bf{r}}}}_{i}\\  & = & \mathrm{2(}Ze{)}^{2}\,\sum _{j\ne i}\,\tfrac{[3({\widehat{{\bf{n}}}}_{ji}\cdot {\widehat{{\bf{r}}}}_{i})({\widehat{{\bf{r}}}}_{j}\cdot {\widehat{{\bf{n}}}}_{ji})-({\widehat{{\bf{r}}}}_{j}\cdot {\widehat{{\bf{r}}}}_{i})]}{4\pi {\epsilon }_{WS}{R}_{ij}^{3}}{r}_{j}(t)=\sum _{j\ne i}\,m{\omega }_{ij}^{2}{\zeta }_{ij}{r}_{j}(t),\end{array}$$

where ${\widehat{{\bf{n}}}}_{ji}=\frac{{{\bf{R}}}_{j}-{{\bf{R}}}_{i}}{{R}_{ij}}$ is the direction joining the electric dipoles and

6 $${\omega }_{ij}^{2}=\frac{2{Z}^{2}{e}^{2}}{4\pi {\epsilon }_{{\rm{W}}{\rm{S}}}m{R}_{ij}^{3}}$$

is a characteristic frequency describing the strength of the dipole-dipole interactions. Moreover

7 $${\zeta }_{ij}=[3({\widehat{{\bf{n}}}}_{ji}\cdot {\widehat{{\bf{r}}}}_{i})({\widehat{{\bf{r}}}}_{j}\cdot {\widehat{{\bf{n}}}}_{ji})-({\widehat{{\bf{r}}}}_{j}\cdot {\widehat{{\bf{r}}}}_{i})]$$

is a geometrical factor depending of the orientation of the electric dipoles and **r**_*j*_(*ω*) is the Fourier Transform of *r*_*j*_(*t*).

### Biological aqueous environment as thermal bath

This work is inspired by the request for observables in real biological systems at molecular level that can detect the presence of long-range electrodynamics interactions among biomolecules. As all biomolecules in real biological environment are in aqueous solution, we have to take into account the presence of surrounding water molecules. Though recent studies reveal that the water in biological system can have a highly non trivial behaviour with respect to electrodynamic fields generated by the electric dipole of biomolecules^9–13^, in this article we will assume the surrounding water to play simply the role of a thermal bath. As a consequence of this, the presence of water molecules can be schematized via the introduction of a stochastic noise (thermal fluctuations) and a viscous friction term (dissipation) in the equation of motion for oscillating electric dipoles. In particular friction viscous forces are due to the aqueous surrounding medium considered as a homogeneous fluid with viscosity *η*_*w*_. We assume that the expression of the viscous force is given by Stokes’ Law acting on each barycentre of electric charge (positive and negative)

8 $${{\bf{F}}}_{{\rm{visc}},i\pm }=-\,\gamma \frac{{{\rm{dr}}}_{i,\pm }}{{\rm{d}}t}\,\,{\gamma }_{i}=6\pi {\eta }_{W} {\mathcal R} $$

where ${\mathcal R}$ is the hydrodynamic radius of a typical biomolecule ($\sim 10{\rm{\AA }}$).

From Eq. (8) it follows that the acceleration on the dipole length is given by

9 $${(m\frac{{{\rm{d}}}^{2}{r}_{i}}{{\rm{d}}{t}^{2}})}_{FR}={(m\frac{{{\rm{d}}}^{2}}{{\rm{d}}{t}^{2}}({{\bf{r}}}_{i,+}-{{\bf{r}}}_{i,-}))}_{FR}\cdot {\widehat{{\bf{r}}}}_{i}=({{\bf{F}}}_{{\rm{visc}},i+}-{{\bf{F}}}_{{\rm{visc}},i-})\cdot {\widehat{{\bf{r}}}}_{i}=-\,\gamma \frac{{{\rm{dr}}}_{i}}{{\rm{d}}t}.$$

On the other hand the stochastic forces are due to the collision of water molecules and freely moving ions on the biomolecules and they correspond to the realization of a thermal bath at temperature *T*. In particular these forces, acting directly on the charge barycentres of each biomolecules, can be described according to the following expression

10 $${{\bf{F}}}_{{\rm{stoch}},i\pm }={\rm{\Xi }}\,{{\boldsymbol{\xi }}}_{i,\pm }(t)\,\,{\rm{\Xi }}=\sqrt{2{k}_{B}T\gamma },$$

where ***ξ***_*i*_(*t*) represents white noise whose characteristics along each Cartesian component *α*, *β* = *x*, *y*, *z* are given by

11 $${\langle {(\xi {(t)}_{i,\pm })}_{\alpha }\rangle }_{t}=0\,{\langle {(\xi {(t)}_{i,\pm })}_{\alpha }{(\xi {(t^{\prime} )}_{j,\pm })}_{\beta }\rangle }_{t}=\delta (t-t^{\prime} ){\delta }_{ij}{\delta }_{\alpha \beta }({\delta }_{++}+{\delta }_{--}-{\delta }_{+-}-{\delta }_{-+})$$

The minus sign in the correlation term is due to the constrain we impose for thermal noise

12 $${{\boldsymbol{\xi }}}_{i,+}(t)=-\,{{\boldsymbol{\xi }}}_{i-}(t\mathrm{).}$$

Such a condition does not take place in general for a real physical system but it has been implemented to provide a consistent realization of a stochastic system such that the center of mass of each molecule is fixed. With this prescription the stochastic force along the dipole direction is given by

13 $${(m\frac{{{\rm{d}}}^{2}{r}_{i}}{{\rm{d}}{t}^{2}})}_{ST}=({{\bf{F}}}_{{\rm{stoch}},i+}(t)-{{\bf{F}}}_{{\rm{stoch}},i-}(t))\cdot {\widehat{{\bf{r}}}}_{i}=2{{\boldsymbol{\xi }}}_{i,+}(t)\cdot {\widehat{{\bf{r}}}}_{i}=2{\rm{\Xi }}{\xi }_{i}(t\mathrm{).}$$

### External forcing to produce out-of-thermal equilibrium conditions

In^5^ it has been shown that long-range interactions among biomolecules can be present if the system of oscillating dipoles is maintained in out-of-thermal equilibrium. To achieve this goal a forcing term *F*_*NE*,*i*_(*t*) has been included in the equations of motion for the electric dipoles in order to ensure an external injection of energy. The explicit form of the force *F*_*NE*,*i*_(*t*) depends on the specific process that is chosen to inject energy into the system. In particular, a possible mechanism that has been used recently in THz spectroscopy experiments to detect collective giant oscillations in biomolecules, is the injection of energy in vibrational modes through the vibrational decay of the excited fluorochromes (i.e. fluorescent dye molecules) attached to each biomolecules^6^. This process can be represented choosing the following explicit form for the forcing term

14 $${F}_{NE,i}(t)={A}_{NE,i}\,{\omega }_{{\rm{p}}{\rm{u}}{\rm{l}}}\,{f}_{{\rm{p}}{\rm{u}}{\rm{l}}}(t;{\omega }_{{\rm{p}}{\rm{u}}{\rm{l}}},{\varphi }_{i})$$

where *f*_pul_ is a pulse-like function of the form

15 $${f}_{{\rm{pul}}}(t;{\omega }_{{\rm{pul}}},{\varphi }_{i})=\frac{1}{2\pi }\sum _{i=1}^{{n}_{{\rm{pul}}}}\,{a}_{n}{[1+\cos ({\omega }_{{\rm{pul}}}t+{\varphi }_{i})]}^{{n}_{{\rm{pul}}}}\,\,{a}_{n}=\frac{{2}^{n}{(n!)}^{2}}{\mathrm{(2}n)!}\mathrm{.}$$

The coefficients in the former equation have been chosen such that the integral of the function *f*_pul_ over a period ${T}_{{\rm{pul}}}=2\pi {\omega }_{{\rm{pul}}}^{-1}$ respects the following normalization

16 $${\int }_{0}^{\frac{2\pi }{{\omega }_{{\rm{pul}}}}}\,{f}_{{\rm{pul}}}(t;{\omega }_{{\rm{pul}}},{\varphi }_{i}){\rm{d}}t=\frac{1}{{\omega }_{{\rm{pul}}}}\,\mathrm{.}$$

With this choice it is clear that *A*_*NE*,*i*_ corresponds to the momentum transferred by the fluorochrome to the protein in a time $2\pi {\omega }_{{\rm{pul}}}^{-1}$. The energy losses in vibrational decay can be estimated to be of the order Δ*E*_pul_ = *h*Δ*ν*_fluor_ where Δ*ν*_*fluor*_ is the difference among frequencies of absorbed and emitted light by the fluorochrome and *h* is the Planck constant; consequently, if *m*_fluor_ is the mass of the fluorochrome, the momentum transferred to the biomolecule can be approximated by

17 $${\rm{\Delta }}({m}_{i}{\dot{r}}_{i})\approx \sqrt{2h{\rm{\Delta }}\nu {m}_{{\rm{f}}{\rm{l}}{\rm{u}}{\rm{o}}{\rm{r}}}}={A}_{NE,i}={A}_{NE}.$$

### Equation of motion for the system of oscillating interacting dipoles

The equations of motion that describe the dynamics of the system with mutually oscillating dipoles are

18 $$\begin{array}{ccc}m\frac{{{\rm{d}}}^{2}{r}_{i}}{{\rm{d}}{t}^{2}} & = & -m{\omega }_{0}^{2}({r}_{i}-{r}_{i0})-m\frac{{\omega }_{0}^{2}}{{\rm{\Lambda }}}{({r}_{i}-{r}_{i0})}^{3}+{\sum }_{j\ne i}\,m{\omega }_{ij}^{2}{\zeta }_{ij}{r}_{j}+\\  &  & -\gamma \frac{{\rm{d}}{r}_{i}}{{\rm{d}}t}+2{\rm{\Xi }}{\xi }_{i}(t)+{F}_{NE,i}(t)\,\,{\rm{\forall }}\,i=1,...,N\end{array}$$

where all the biomolecules are assumed to be identical so that they all have the same characteristic frequencies *ω*_*i*_ = *ω*_0_ and Λ_*i*_ = Λ.

In order to simplify the discussion we rescale the system according to

19 $$m=\mu \tilde{m},\,\,t=\frac{\tau }{{\omega }_{0}},\,\,{r}_{i}=\lambda {x}_{i},\,\,\frac{{\rm{d}}{r}_{i}}{{\rm{d}}t}=\lambda {\omega }_{0}\frac{{\rm{d}}{x}_{i}}{{\rm{d}}\tau }$$

that substituted in Eq. (18) give the following system of stochastic differential equations of first order

20 $$\{\begin{array}{cc}\frac{{\rm{d}}{x}_{i}}{{\rm{d}}\tau }\,=\, & {\upsilon }_{i}\\ \frac{{\rm{d}}{\upsilon }_{i}}{{\rm{d}}\tau }\,=\, & -({x}_{i}-{x}_{i0})-\frac{{({x}_{i}-{x}_{i0})}^{3}}{{\mathop{{\rm{\Lambda }}}\limits^{ \sim }}^{2}}-{{\rm{\Omega }}}_{{\rm{f}}{\rm{r}}{\rm{i}}{\rm{c}}{\rm{t}},i}\frac{{\rm{d}}{x}_{i}}{{\rm{d}}\tau }+\sum _{j\ne i}^{N}\,{{\rm{\Omega }}}_{ij}^{2}{\zeta }_{ij}{x}_{j}+{\mathop{{\rm{\Psi }}}\limits^{ \sim }}_{i}{\mathop{\xi }\limits^{ \sim }}_{i}(t)\\  & +\,{{\rm{\Omega }}}_{{\rm{p}}{\rm{u}}{\rm{l}}}{{\mathscr{A}}}_{NE}\,{f}_{{\rm{p}}{\rm{u}}{\rm{l}}}(\tau ;{{\rm{\Omega }}}_{{\rm{p}}{\rm{u}}{\rm{l}}},{\varphi }_{i})\,{\rm{\forall }}\,i=1,...,N\end{array}$$

where

21 $$\begin{array}{c}\mathop{{\rm{\Lambda }}}\limits^{ \sim }=\frac{{\rm{\Lambda }}}{\lambda },\,\,{{\rm{\Omega }}}_{ij}^{2}=\frac{{\omega }_{ij}^{2}}{{\omega }_{0}^{2}},\,\mathop{{\mathscr{R}}}\limits^{ \sim }=\frac{{\mathscr{R}}}{\lambda },\,{\mathop{\eta }\limits^{ \sim }}_{W}=\frac{{\eta }_{W}\lambda }{\mu {\omega }_{0}},\,{{\rm{\Omega }}}_{{\rm{f}}{\rm{r}}{\rm{i}}{\rm{c}}{\rm{t}},i}=\frac{6\pi \mathop{{\mathscr{R}}}\limits^{ \sim }{\mathop{\eta }\limits^{ \sim }}_{W}}{{\mathop{m}\limits^{ \sim }}_{i}},\,{{\mathscr{E}}}_{{\rm{b}}{\rm{a}}{\rm{t}}{\rm{h}}}=\frac{{k}_{B}T}{\mu {\lambda }^{2}{\omega }_{0}^{2}},\\ {\mathop{\xi }\limits^{ \sim }}_{i}={\omega }_{0}^{-1/2}{\xi }_{i},\,{\mathop{{\rm{\Psi }}}\limits^{ \sim }}_{i}={(\frac{48\pi {{\mathscr{E}}}_{{\rm{b}}{\rm{a}}{\rm{t}}{\rm{h}}}\mathop{{\mathscr{R}}}\limits^{ \sim }{\mathop{\eta }\limits^{ \sim }}_{W}}{{\mathop{m}\limits^{ \sim }}_{i}^{2}})}^{1/2},\,{{\rm{\Omega }}}_{{\rm{p}}{\rm{u}}{\rm{l}}}=\frac{{\omega }_{{\rm{p}}{\rm{u}}{\rm{l}}}}{{\omega }_{0}},\,{{\mathscr{E}}}_{{\rm{p}}{\rm{u}}{\rm{l}}}=\frac{h{\rm{\Delta }}{\nu }_{{\rm{f}}{\rm{l}}{\rm{u}}{\rm{o}}{\rm{r}}{\rm{r}}}}{\mu {\omega }_{0}^{2}{\lambda }^{2}},\\ {\mathop{m}\limits^{ \sim }}_{{\rm{f}}{\rm{l}}{\rm{u}}{\rm{o}}{\rm{r}}}=\frac{{m}_{{\rm{f}}{\rm{l}}{\rm{u}}{\rm{o}}{\rm{r}}}}{\mu },\,\,{{\mathscr{A}}}_{NE}={(\frac{{{\mathscr{E}}}_{{\rm{p}}{\rm{u}}{\rm{l}}}{\mathop{m}\limits^{ \sim }}_{fluor}}{{\mathop{m}\limits^{ \sim }}_{i}^{2}})}^{1/2}.\end{array}$$

### Choice of numerical parameters in eq. (20)

The numerical values of parameters that appear in Eq. (20) have been estimated for a realistic biological system. In particular the characteristic fundamental scales for the system have been fixed as following: (i) the typical mass scale of a biomolecule *μ* = 1.66 × 10^−24^ Kg = 1 KDa; (ii) the characteristic length scale of a biomolecule *λ* = 10^−9^ *m*; (iii) the characteristic frequency of the collective oscillations for a biomolecule *ω*_0_ = 10^12^ *s*^−1^. Moreover, since we would test the eventual emergence of self-organized synchronization, we consider a set of identical molecules in order to maximise the probability of observing it; therefore we assume ${\tilde{ {\mathcal R} }}_{i}=1$, ${\tilde{m}}_{i}=10$ and ${x}_{i0}={r}_{i,eq}/\lambda \simeq 5$ for all *i* = 1, … *N* according to characteristic dimension and masses of biomolecules.

The parameter that fixes the characteristic length for the emergence of non linear phenomena has been settled to be $\tilde{\Lambda }\simeq 0.85$. The temperature of the system has been settled at *T* = 300 *K* and consequently for our choices ${{\mathscr{E}}}_{{\rm{b}}{\rm{a}}{\rm{t}}{\rm{h}}}=2.5\,\times \,{10}^{-3}$, while water viscosity is ${\eta }_{W}\simeq 8.54\times {10}^{-4}{\rm{Pa}}\cdot {\rm{s}}$ and ${\tilde{\eta }}_{W}=0.514$ yielding Ω_frict,*i*_ = Ω_frict_ = 0.97. With our choice of free parameters of the system, the strength of thermal noise results $\tilde{{\rm{\Psi }}}\simeq 4.4\times {10}^{-2}$. The frequencies associated to the electrodynamic interactions ${{\rm{\Omega }}}_{ij}^{2}$ can be expressed in terms of adimensionalized units

22 $${{\rm{\Omega }}}_{ij}^{2}=\frac{1}{{\omega }_{0}^{2}}\frac{2{e}^{2}}{4\pi {\epsilon }_{WS}\mu {\lambda }^{3}}\frac{{Z}^{2}}{\tilde{m}{\tilde{R}}_{ij}^{3}}=\frac{1}{{\omega }_{0}^{2}}\frac{2{e}^{2}}{4\pi \,{\epsilon }_{WS}\,\mu {\lambda }^{3}}\frac{{Z}^{2}}{\tilde{m}{\tilde{R}}_{ij}^{3}}$$

where ${\tilde{R}}_{ij}$ is the mutual distance between the centers of the dipoles *i* and *j* expressed in unit of *λ* and $\tilde{m}$ is the mass of a molecule expressed in adimensionalized units. In the performed simulations the position of each dipole representing a biomolecule is assigned in a cube box of side $l={N}^{\mathrm{1/3}}\langle \tilde{d}\rangle$, i.e. the components of the vector position of the center of each dipole have coordinates ${\tilde{{\bf{R}}}}_{i}=l{{\bf{x}}}_{{R}_{i}}=l({x}_{{R}_{i}},{y}_{{R}_{i}},{z}_{{R}_{i}})$, with ${x}_{{R}_{i}},{y}_{{R}_{i}},{z}_{{R}_{i}}\in [0,1]$, where *N* is the total number of dipoles and $\langle \tilde{d}\rangle$ is the average intermolecular distance in *λ* units. As a reference case in our simulations the parameters have been chosen to be $\tilde{m}=10$, *Z*_*i*_ = 1000, while the average intermolecular distance $\langle \tilde{d}\rangle =\lambda \langle \tilde{d}\rangle =1.6\times {10}^{3}{\rm{\AA }}=1.6\times {10}^{-7}m$. The reason for choosing such a large value of *Z* is justified under the hypothesis that the surrounding water molecules participate to the effective dipole of each biomolecule and enhance it^5,9^. Therefore for the considered choice of parameters ${{\rm{\Omega }}}_{ij}^{2} \sim 2.3\times {10}^{-3}$. Finally, in order to consider different cases with stronger interactions (corresponding to shorter average intermolecular distances, for instance) the coupling term is multiplied by a factor *K* > 0 with respect to the reference case just discussed, i.e.

23 $${{\rm{\Omega }}}_{ij}^{2}=\frac{1}{{\omega }_{0}^{2}}\frac{2{e}^{2}}{4\pi {\epsilon }_{WS}\mu {\lambda }^{3}}\frac{{Z}^{2}}{\mathop{m}\limits^{ \sim }N{\langle \mathop{d}\limits^{ \sim }\rangle }^{3}|{{\bf{x}}}_{{R}_{i}}-{{\bf{x}}}_{{R}_{j}}|}=K{{\rm{\Omega }}}_{ij}^{2}{|}_{\langle \mathop{d}\limits^{ \sim }\rangle =1.6\times {10}^{2}}=K{{\rm{\Omega }}}_{ij,ref}^{2}$$

and by introducing Eq. (23) in Eq. (20) we obtain

24 $$\begin{array}{ccc}\frac{{\rm{d}}{x}_{i}}{{\rm{d}}\tau } & = & {\upsilon }_{i}\\ \frac{{\rm{d}}{\upsilon }_{i}}{{\rm{d}}\tau } & = & -({x}_{i}-{x}_{i0})-\frac{{({x}_{i}-{x}_{i0})}^{3}}{{\mathop{{\rm{\Lambda }}}\limits^{ \sim }}^{2}}-{{\rm{\Omega }}}_{{\rm{f}}{\rm{r}}{\rm{i}}{\rm{c}}{\rm{t}},i}\frac{{\rm{d}}{x}_{i}}{{\rm{d}}\tau }+K\,{\sum }_{j\ne i}^{N}\,{{\rm{\Omega }}}_{ij,ref}^{2}{\zeta }_{ij}{x}_{j}+{\mathop{{\rm{\Psi }}}\limits^{ \sim }}_{i}{\mathop{\xi }\limits^{ \sim }}_{i}(t)\\  &  & +\,{{\rm{\Omega }}}_{{\rm{p}}{\rm{u}}{\rm{l}}}{{\mathscr{A}}}_{NE}\,{f}_{{\rm{p}}{\rm{u}}{\rm{l}}}(\tau ;{{\rm{\Omega }}}_{{\rm{p}}{\rm{u}}{\rm{l}}},{\varphi }_{i})\,\,{\rm{\forall }}\,i=1,\ldots ,N\end{array}$$

This paper is intended as a first feasibility study for the detection of long-range electromagnetic interactions amongs biomolecules in aqueous solutions via a spectroscopic observable. In laboratory conditions the only parameter concerning molecule space configuration and orientation that one can easily control, is the biomolecules concentration, i.e. the intermolecular average distance. Therefore we investigate the emergence of collective behavior between biomolecules by varying the average distance among dipoles that corresponds in our model Eq. (24) to vary K. On the other hand, in this work we do not investigate the role played by spatial correlation of the position and orientation of the dipoles in the appearance of a spectroscopic signature of the long-range electrodynamic dipole-dipole interactions. The study of dependence of spectroscopic observables on spatial correlations could be very interesting in this framework but we postpone to future work this investigation.

The parameter ${{\mathscr{E}}}_{{\rm{p}}{\rm{u}}{\rm{l}}}$ can be estimated assuming that the energy injection on each biomolecule is due to the vibrational decay of a fluorescent dye. It is realistic^6^ to consider a difference between the absorbed and emitted frequency of the order of ${\rm{\Delta }}{v}_{{\rm{fluor}}}\simeq 5\times {10}^{13}{{\rm{s}}}^{-1}$ and ${\tilde{m}}_{{\rm{fluor}}}\simeq 0.6$, thus yielding ${{\mathscr{A}}}_{NE}\simeq 1.4\times {10}^{-2}$.

Finally, the characteristic frequency for the energy transfer Ω_pul_ is one of the most delicate parameters to be settled. This term accounts for the continuous injection of energy into the system, however the release must be done without perturbing too much the oscillating behavior, therefore we can assume that ${{\rm{\Omega }}}_{i}\gg {{\rm{\Omega }}}_{{\rm{pul}}}\simeq {10}^{-2}$.

## Numerical Results

The reported analyses have been done using a single system size (N = 50) and random initial conditions both for positions and velocities. However, similar results have been obtained for N = 100, 200 (not shown). The collective evolution of the population and in particular the level of coherence is usually characterized in terms of the macroscopic field

25 $$\rho (t)={r}_{1}(t){e}^{i{\rm{\Phi }}(t)}=\frac{1}{N}\sum _{j=1}^{N}\,{e}^{i{\theta }_{j}(t)},$$

where the modulus *r*_1_ is an order parameter for the synchronization transition being one (${\mathscr{O}}({N}^{-\mathrm{1/2}})$) for synchronous (asynchronous) states, while Φ is the phase of the macroscopic indicator^14,15^. However, in our case, the molecules are pivoted to the center of mass and cannot rotate: the effective degree of freedom of these objects consists in an elongation/shrinkage along the direction identified by the mutual distance between the two centers of charges. Therefore it is not possible to describe the movement of the dipole in terms of an oscillator rotating along the unit-circle via the identification of a time-dependent phase. The solution that we have adopted is to calculate the phase of the single molecule by using the inversion formulas

26 $$\sin \,{\theta }_{i}=\frac{{x}_{i}-{x}_{0i}}{\sqrt{{\upsilon }_{i}^{2}+{({x}_{i}-{x}_{0i})}^{2}}},\,\,\cos \,{\theta }_{i}=\frac{{\upsilon }_{i}}{\sqrt{{\upsilon }_{i}^{2}+{({x}_{i}-{x}_{0i})}^{2}}}$$

to associate a phase *θ*_*i*_ ∈ [−*π*, *π*] according to

27 $$\begin{array}{c}{\theta }_{i}=\{\begin{array}{cc}\arcsin (\sin \,{\theta }_{i}) & \,{\rm{i}}{\rm{f}}\,\cos \,{\theta }_{i}\ge 0\\ \pi -\arcsin (\sin \,{\theta }_{i}) & \,{\rm{i}}{\rm{f}}\,\sin \,{\theta }_{i} > 0\wedge \,\cos \,{\theta }_{i} < 0\\ -\pi -\arcsin (\sin \,{\theta }_{i}) & \,{\rm{i}}{\rm{f}}\,\sin \,{\theta }_{i} < 0\wedge \,\cos \,{\theta }_{i} < 0.\end{array}\end{array}$$

**Figure 1:**
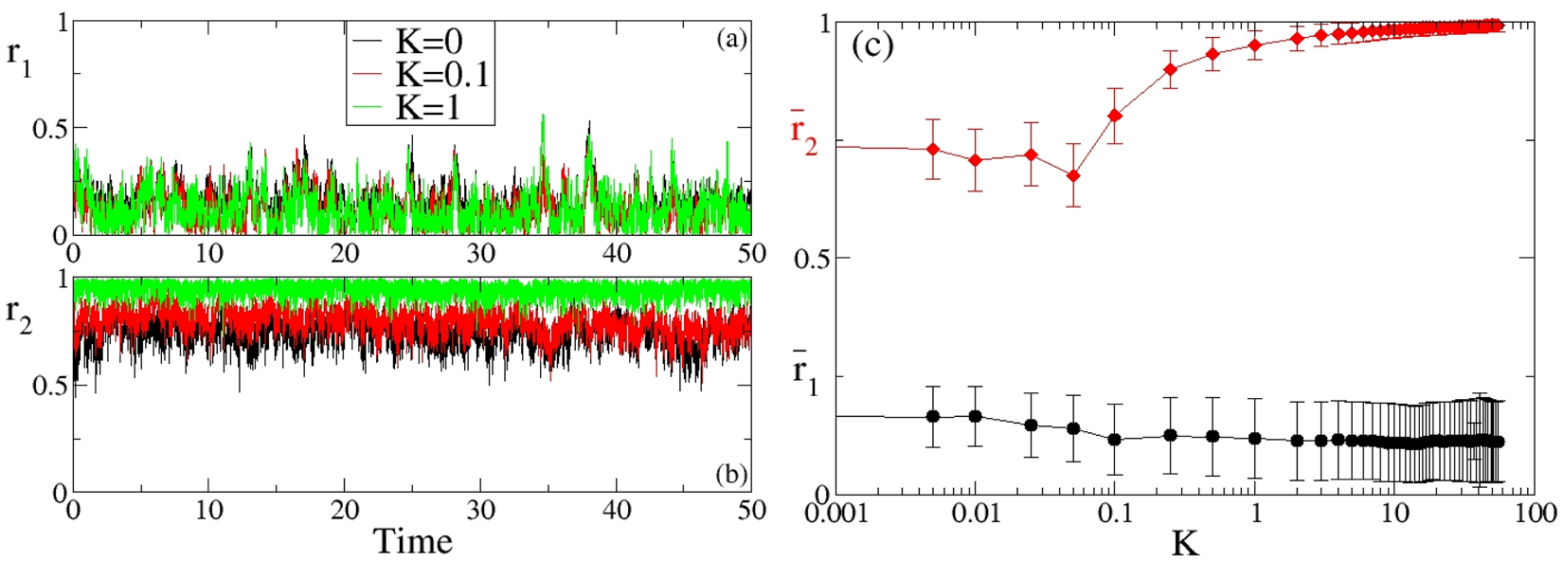
Synchronization properties of the system. Order parameters *r*_1_ (**a**), *r*_2_ (**b**) as a function of time for different coupling constants. Panel (c) Time-averaged order parameters as a function of the coupling constant K. The parameters values used for these simulations are: Ω_*i*_ = 0.01, *x*_*i*0_ = 5, Ω_*frict*,*i*_ = 0.97 (for every *i* = 1, …, *N*), Ω_*pul*_ = 0.1, ${{\mathscr{A}}}_{NE}=0.011$, *N* = 50.

**Figure 2:**
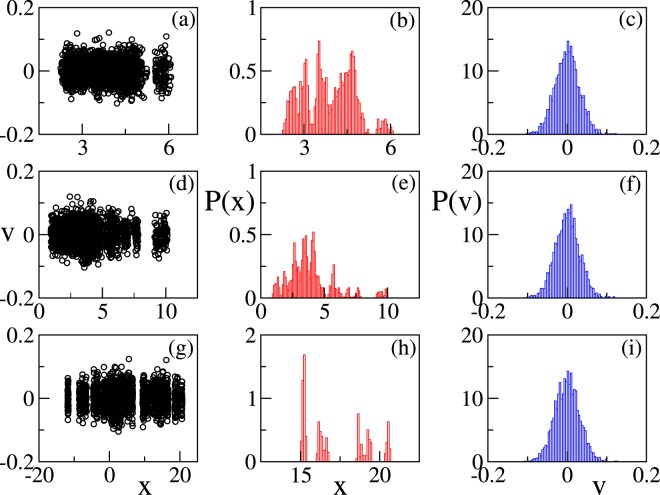
Panels (a,d,g) Snapshots of the velocities of the single dipoles as a function of their positions for K = 0 (**a**), K = 0.1 (**d**), K = 1 (**g**). Panels (b,e,h) probability distribution of the positions of the dipoles for different coupling constants. The panels refer to K = 0 (b), K = 0.1 (**e**), K = 1 (**h**). Panels (c,f,i) probability distribution of the velocities of the dipoles for different coupling constants. The panels refer to K = 0 (**c**), K = 0.1 (**f**), K = 1 (**i**). The probability distribution functions are obtained by measuring the corresponding variables at regular time intervals *δT* during a long simulation, after discarding an initial transient time. Parameters as in Fig. 1.

**Figure 3:**
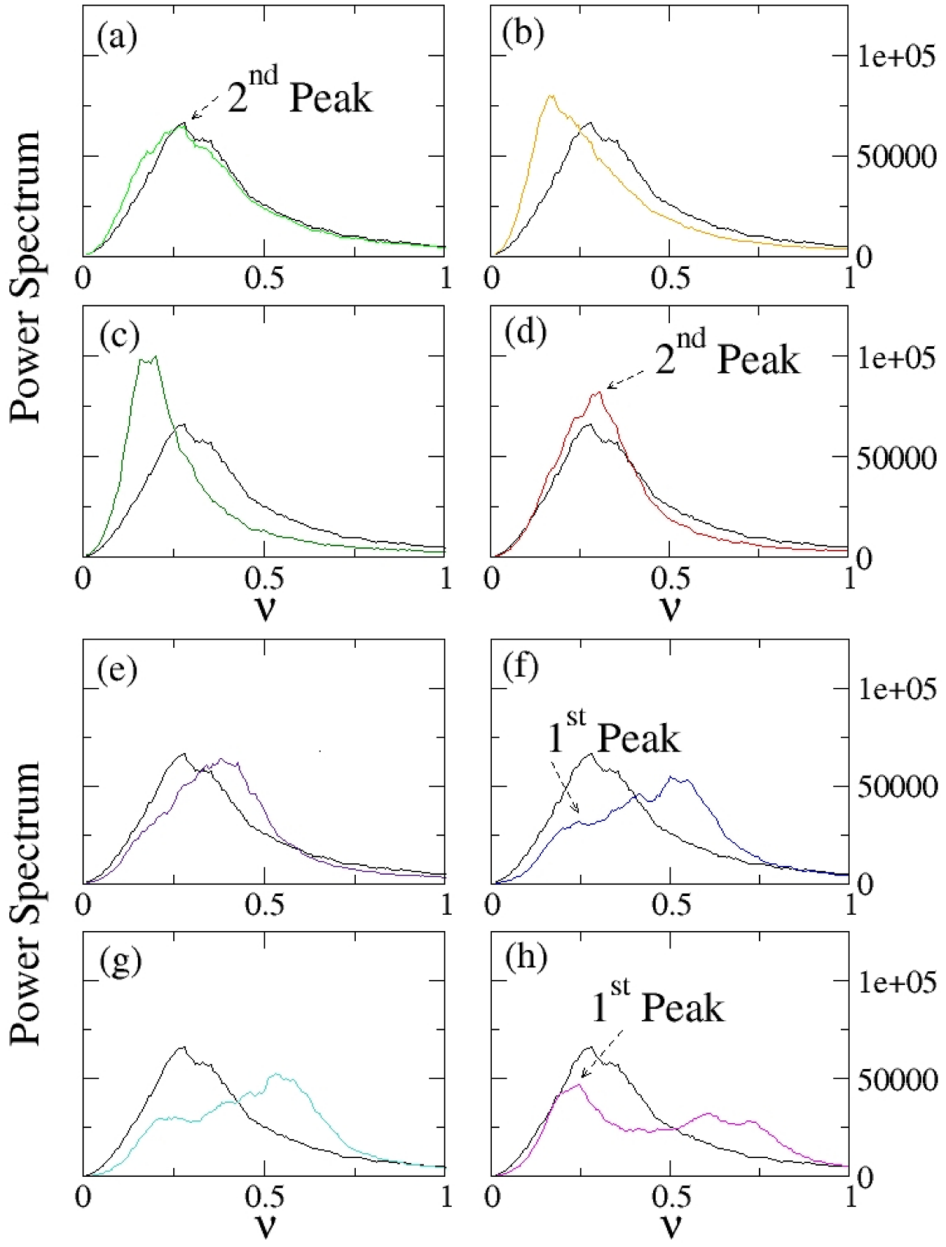
Investigation of the emergence of a collective behavior as a characteristic peak in the power spectrum. Panels (a–h) Power spectrum of *dP*/*dt* for different values of the coupling constant K and for thermal noise strength ${\tilde{{\rm{\Psi }}}}_{{\rm{i}}}$ = 0.044. The black curve represents, in each panel, the power spectrum of the system without coupling (K = 0). The other curves shown are, respectively, for *K* = 0.1 (**a**); *K* = 0.25 (**b**); *K* = 1 (**c**), *K* = 5 (**d**); *K* = 10 (**e**); *K* = 19 (**f**); *K* = 21 (**g**); *K* = 31 (**h**). The parameters values used for these simulations are: Ω_*i*_ = 0.01, *x*_*i*0_ = 5, Ω_*frict*,*i*_ = 0.97 (for every *i* = 1, …, *N*), Ω_*pul*_ = 0.1, ${{\mathscr{A}}}_{NE}=0.011$, *N* = 50.

**Figure 4:**
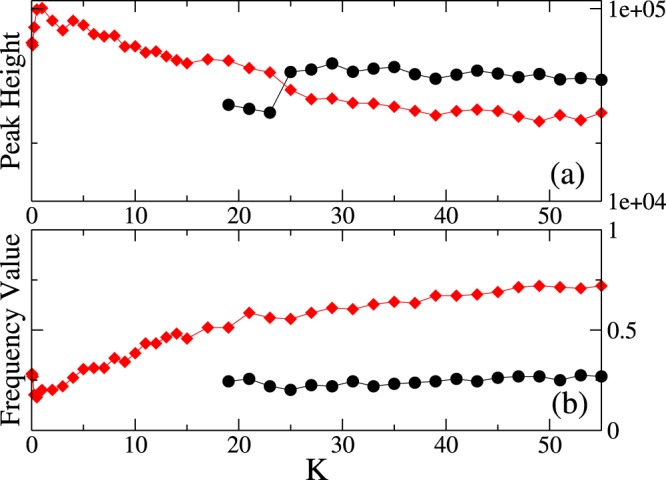
Dependence of the system’s characteristic frequencies on the coupling constant. Panels (a,b): Peak height (**a**) and frequency value (**b**) of the first two main peaks that characterize the dynamics of the system. Red diamonds identify the primary peak, black dots the secondary one. Parameters as in Fig. 3.

In the following we present two set of parameters: a first one corresponding to the values discussed in Sec. II F and a second one, where we arbitrarily increase the thermal noise to investigate the robustness of the system and the emergent collective effects. The first set of parameters has been used to find the results shown in Figs (1–4). In this case the calculation of the order parameter *r*_1_ does not lead to the identification of emergent (phase) synchronization in the system; in particular *r*_1_ does not show any dependence on the coupling constant (see Fig. 1, panels (a) and (c)), as we would expect when the molecules are interacting with increasing strength. On the other hand if we calculate the order parameter usually employed to identify the emergence of 2-clusters $({r}_{2}(t)=|\frac{1}{N}{\sum }_{j\mathrm{=1}}^{N}\,{e}^{i2{\theta }_{j}(t)}|)$, we clearly observe a transition to cluster synchronization already for small coupling constants (see Fig. 1(c)). This is confirmed if we calculate the distribution of positions and velocities of the molecules (see Fig. 2, panels (a–i)). In particular, if we look at the phase space (*x*, *v*) it emerges clearly that the system splits naturally in 2 clusters and the distance between the clusters increases if the single elements are coupled. For stronger coupling more clusters emerge leading to a partial cluster synchronization. Moreover the probability distribution functions of the positions reveal an increase of the elongation towards values that turn out to be not realistic from a bio-physical point of view if the coupling constant is too big $(K\gg 1)$. Only the probability distribution profile of the velocities remains unchanged if the coupling constant and, therefore, the distance among the dipoles, is changed.

A better insight of the emergence of a collective behavior can be achieved if we consider the total dipole moment

28 $$P(t)=\sqrt{\sum _{i=1}^{N}\,{\Vert {{\bf{x}}}_{i}(t)-{{\bf{x}}}_{i\mathrm{,0}}\Vert }^{2}}$$

with ***x***_*i*_ = *x*_*i*_(cos *ϕ*_*I*_ sin *β*_*i*_, sin *ϕ*_*I*_ sin *β*_*i*_, cos *β*_*i*_). *P*(*t*) represents the ensemble average of the projection of the dipole position in the cartesian coordinates system *X*, *Y*, *Z*. The biomolecule in our model is identified via the intermolecular mutual distance between the two centers of charges measured along the radial *x* direction and we need to express this variable in cartesian coordinates. In other words, for each molecule we have projected the dipole position along the directions *X*, *Y*, *Z*, thanks to the respective projection angle *β*_*i*_ of each molecule’s radius to the Z-axis and *ϕ*_*i*_, projection angle of *x*_*i*_ to the X-axis in the XY plane. These angles are generated together with the initial conditions and do not vary in time.

Due to the fact that the system is not deterministic and a white noise source is present into the differential equations, we have developed a method similar to the second-order Runge-Kutta one for solving numerically ordinary differential equations. In particular we have implemented the Heun method^16^ in the Runge-Kutta algorithm as suggested in^17^, and we have used an integration time step 0.002 to perform the simulations. In addition to this, in order to compare the results for different coupling constants and for different strengths of the thermal noise, we implemented a low-pass filter to analyse the power spectra. This filter relies on the differentiation properties of the Fourier transform; in particular, since the Fourier transform of a generic function *f* is related to the Fourier transform of its derivative via the relationship ${\mathcal F} \,[\,f^{\prime} (\nu )]=2\pi iv\widehat{f}(v)$, it is possible to filter the low-frequency components of the spectrum just using the Fourier transform of the derivative. The low-frequency components that we want to filter out are related to the injected white noise that are not interesting for the scope of this work, i.e. finding a mark of emergent collective behavior.

Therefore we calculated the power spectrum of *dP*/*dt* to investigate the role played by the interactions among the dipoles to enhance a collective motion. While non-coupled dipoles show a peak at frequency ≈0.280 ± 0.006 (Fig. 3(a)), as soon as a small coupling is present in the system, the interactions among the dipoles get stabilized and a peak at lower frequency emerges already for *K* = 0.25 (Fig. 3(b)). However the eigenmodes emerging for small coupling are destroyed for bigger coupling, where the non-linearity in Eq. (24) prevents this self-organized behavior at small frequencies (Fig. 3(e)). The peak at small frequency emerges again for *K* ≥ 19 (Fig. 3(f)), but the intensity shown is smaller than before. On the other hand the main peak moves to higher frequencies for increasing coupling, but the intensity is more and more depressed (Fig. 3(g,h)).

A summary is presented in Fig. (4), where the peak intensity and the corresponding frequency values are given as a function of the coupling; while the secondary peak emerging at low frequency value for *K* ≥ 19 remains almost stable and constant, the primary peak, already visible for small coupling (*K* = 0.25) changes its form and shifts towards higher frequency values.

**Figure 5:**
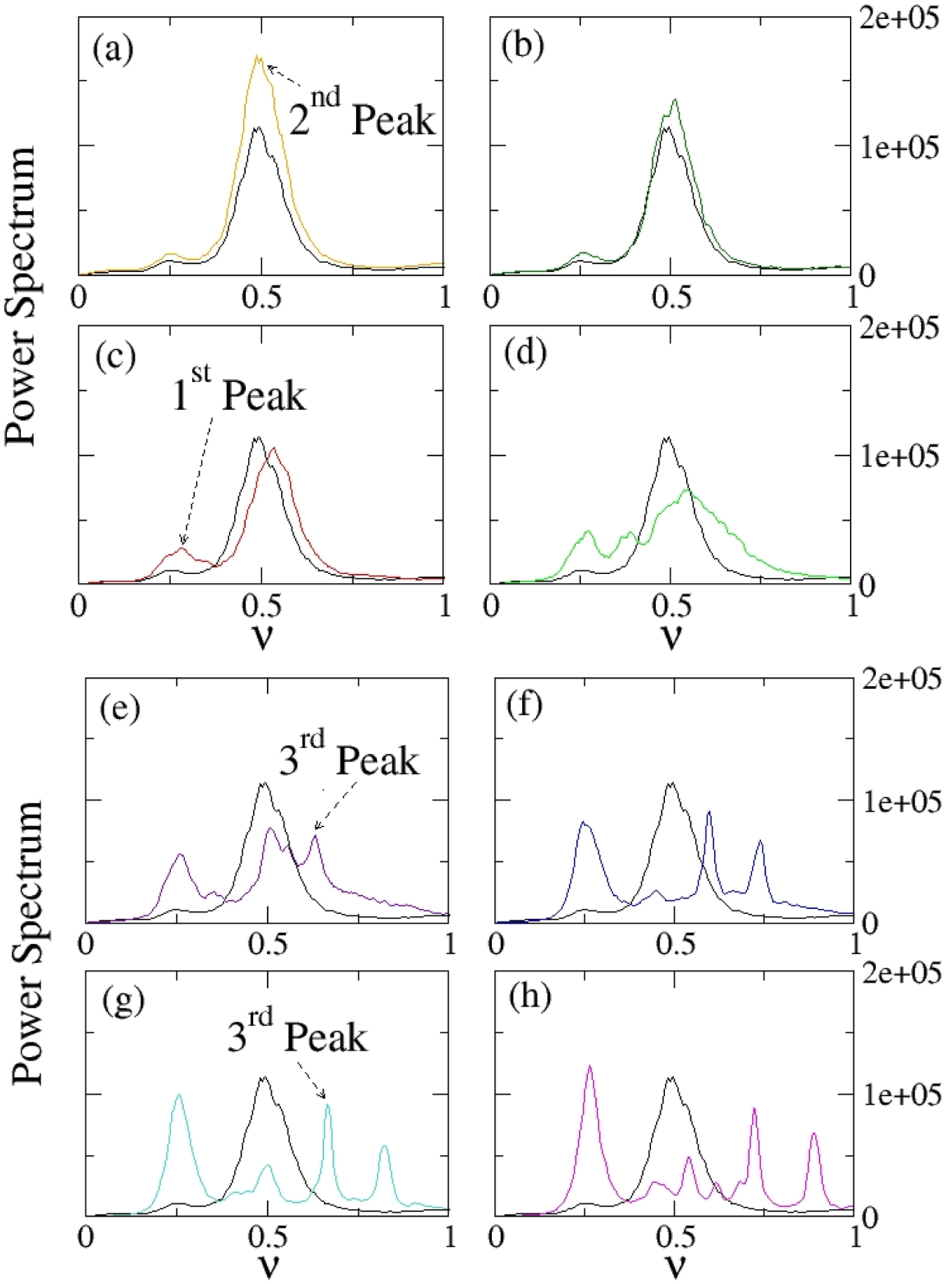
Investigation of the emergence of a collective behavior as a characteristic peak in the power spectrum. Panels (a–h) Power spectrum of *dP*/*dt* for different values of the coupling constant K and for thermal noise strength ${\tilde{{\rm{\Psi }}}}_{i}$ = 0.46. The black curve represents, in each panel, the power spectrum of the system without coupling (K = 0). The other curves shown are, respectively, for *K* = 1 (**a**); *K* = 2 (**b**); *K* = 5 (**c**), *K* = 10 (**d**); *K* = 21 (**e**); *K* = 31 (**f**); *K* = 41 (**g**); *K* = 50 (**h**). The parameters values used for these simulations are: Ω_*i*_ = 1, *x*_*i*0_ = 5, Ω_*frict*,*i*_ = 0.105 (for every *i* = 1, …, *N*), Ω_*pul*_ = 0.1, ${{\mathscr{A}}}_{NE}=1.4$, *N* = 50.

**Figure 6:**
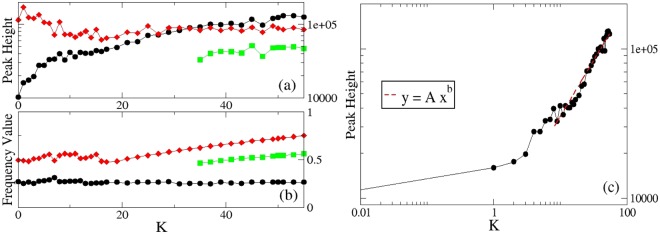
Dependence of the system’s characteristic frequencies on the coupling constant. Panels (a,b) Peak height (**a**) and frequency value (**b**) of the first three main peaks that characterize the dynamics of the system. Panel (c) Fitting of the dependence of the peak height on the coupling constant. Fitting values are *A* = 6188, 4 ± 0.5, *b* = 0.75 ± 0.03. For all the panels the black dotted curve represents the first peak, the red diamonds curve represents the second peak and the square green curve represents the third peak. Parameters as in Fig. 5.

The results for the second set of parameters are given in the Figs (5 and 6). We refer to the Supplementary Information (Sec. II) for the analysis of the synchronization level for this second set of parameters. While in absence of interactions (K = 0), the system shows a single pronounced peak at frequency ≈0.488 ± 0.006, once the interactions are active (*K* > 0), another peak arises at smaller frequency ≈0.263 ± 0.013. By increasing the value of K we observe an increase of the peak at lower frequency, to which corresponds a decreasing of the peak at higher frequency: a collective motion is enhanced due to interaction, while the motion corresponding to the non-connected situation is depressed (see Figs 5, panels (a–h) and 6(a)). On the other hand the position of the peak (i.e. the corresponding frequency value) does not change significantly if we increase the coupling constant (see Fig. 6(b)); the more evident increasing ratio for *K* > 20 is related to the fact that power spectra become richer and richer for higher coupling and secondary peaks arise. One of these secondary peaks (the main one) emerging at bigger coupling constant is also reported in Fig. 6 (panels (a,b)), and it is termed “Third Peak”.

Finally, if we analyze in more detail the behavior of the first peak, related to the emergent collective motion, as a function of the coupling constant, it is possible to identify two different scales, once the figure is plotted in log-log scale (Fig. 6(c)). In particular, the different scales present for low coupling constant (*K* < 5) and for sufficiently strong coupling (*K* > 10) denote a transition between two different dynamical behaviors: the cross-over between two different regimes, from the one dominated by individual asynchronous behavior, to the one dominated by collective motion, with strongly interacting oscillators, is thus compatible with these two different scales.

**Figure 7:**
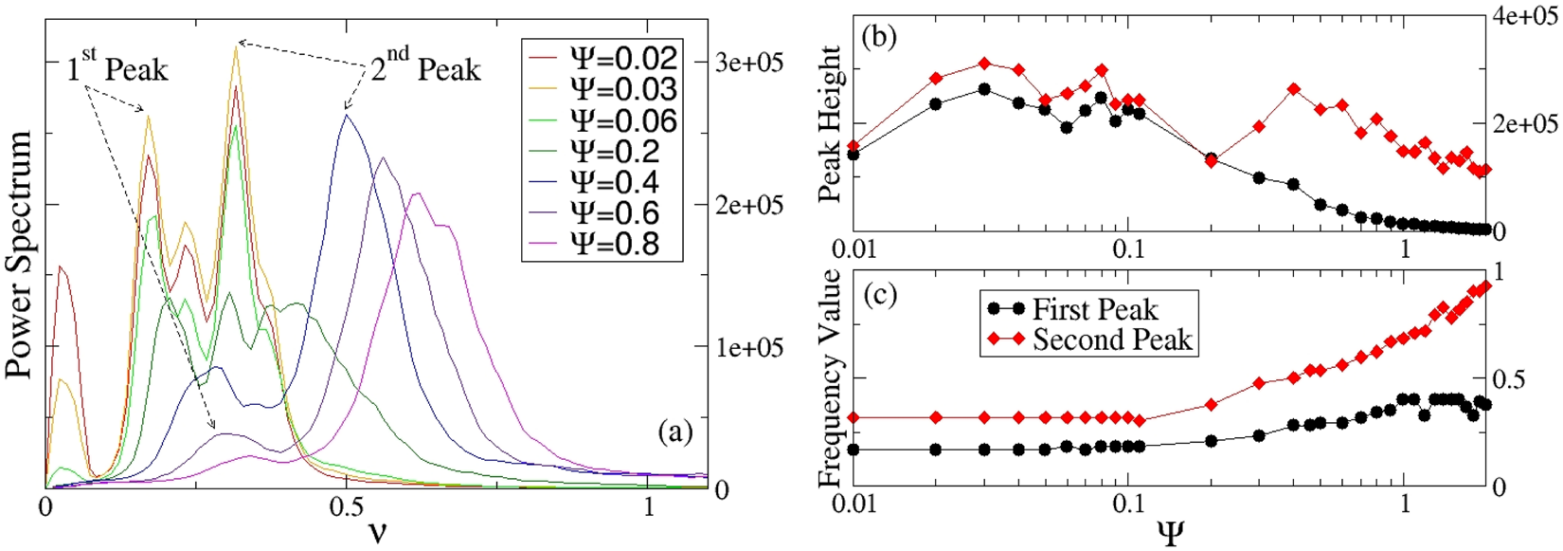
Response of the system under the effect of the thermal noise strength. Panel (a) Power spectrum of *dP*/*dt* for different values of the thermal noise strength and for coupling constant K = 5. Panels (b,c): Peak height (**b**) and frequency value (**c**) of the first two main peaks that characterize the dynamics of the system. Parameters as in Fig. 5. The values of the different thermal noise strengths reported in the caption of panel (a) and the axis label in panel (c) must be intended as $\tilde{{\rm{\Psi }}}$: the ^~^ has been suppressed in the figure for the sake of simplicity.

If we now investigate the role of the thermal noise strength, we obtain a stochastic resonance effect^18^: the signal at low frequency (≈0.28 ± 0.09) can be boosted by adding white noise to the signal, which contains a wide spectrum of frequencies. The frequencies in the white noise spectrum corresponding to the original signal’s frequencies resonate with each other, thus amplifying the original signal (i.e. the signal at low frequency) while not amplifying the rest of the white noise. Furthermore the signal-to-noise ratio is increased, while the added white noise is filtered out thanks to the band-pass filter that we have implemented calculating the power spectrum of *dP*/*dt*. In particular the low frequency peak, that corresponds in our case to the collective motion, is more visible for thermal noise strength $\tilde{{\rm{\Psi }}}$ = 0.03, to which corresponds a maximum in the peak high (see Fig. 7 panels (a,b)). This peak is depressed for higher temperature and less likely to be revealed. On the other hand the peak at high frequency (≈0.56 ± 0.22), corresponding to the dynamics of isolated dipoles, can be also boosted by adding white noise into the system, but it does not decrease as significantly as the former one for higher temperatures, thus meaning that the single dipoles in this model are able to react to big level of noise, even though this is physically not plausible, since we would expect that dipoles will break up for high temperatures.

## Discussion

Let us now comment about the physical meaning, and about the prospective relevance, of the results described in the previous Sections. As repeatedly stated, the present study was motivated by the need of finding an experimental strategy - complementary to the diffusion-based one already discussed in^3–5^ - to detect the possible presence of electrodynamic attractive forces between biomolecules. Such a possibility emerges in the following framework. By pumping energy in the biomolecules of an aqueous solution, that is by keeping these molecules warmer than the solvent (out-of-thermal equilibrium), when the input energy rate exceeds a threshold value, then all, or almost all, the excess energy (that is, energy input minus energy losses due to dissipation) is channeled into the vibrational mode of the lowest frequency. In other words, the shape of the entire molecule is periodically deformed resulting in a “breathing” movement^6^. In doing so the biomolecules behave as microscopic antennas that absorb the electromagnetic radiation tuned at their “breathing” (mesoscopic) oscillation frequency. But antennas at the same time absorb and re-emit electromagnetic radiation, thus, according to a theoretical prediction, these antennas (biomolecules) can attractively interact at a large distance through their oscillating near-fields, and through the emitted electromagnetic radiation, provided that these oscillations are resonant and thus, take place at the same frequency^6^. The still open question is whether these electrodynamic interactions can be strong enough to be experimentally detectable, which would mean, in the positive case, to have some prospective biological relevance.

In the model of an aqueous solution of biomolecules that we have tackled, each individual molecule is assumed to be driven to an out-of-equilibrium mesoscopic vibrational mode which, in turn, excites an attractive electrodynamic force field associated with a −1/*r*^3^ potential, where *r* is the intermolecular distance. By setting the parameters of the model to physically realistic values, we have numerically investigated the effect of varying the strength *K* of the mutual dipole-dipole electrodynamic interaction. The new observed phenomenon, shown in Section III, is the appearance of a collective behaviour involving all the molecules of the system, which is identified through *K*-dependent spectral features of a suitable macroscopic observable *P*(*t*), defined in Eq. (28). Furthermore, the analysis of the level of coherence in the system, done in terms of the standard Kuramoto order parameter *r*_1_ and of the 2-cluster order parameter *r*_2_, shows that the collective dynamics cannot be simply traced back to synchronization. In particular, the order parameter *r*_1_ measures the level of synchronization, while *r*_2_ measures the degree of cluster synchrony in the system. Both order parameters oscillate irregularly thus implying collective irregular dynamics, irrespectively of the regular, periodic (breathing) behavior of the single dipole units (see also Fig. 4(a,b) in the Supplementary Information). Due to the combined effect of an external periodical forcing, of the presence of noise and of the interactions among biomolecules, we observed a self-organized formation of phase clusters characterized by different velocities and leading to (possibly) chaotic or quasiperiodic behavior at the macroscopic level. The emergence of nontrivial collective dynamics in systems composed of elements whose evolution is extremely simple, is a well-known phenomenon observed in complex systems. In particular chaotic irregular behavior emerging from regular unit dynamics has been seen in^19^, while the emergence of quasiperiodic motion has been shown in systems of oscillators^20^, neuron models^21^ and rotators^22^.

From an experimental point of view, this means that by performing spectroscopic measurements at different concentrations of the solvated biomolecules we could detect the presence of electrodynamic intermolecular interactions. Varying the concentration *C* of the solution entails the variation of the average intermolecular distance 〈*d*〉 according to the relation 〈*d*〉 = *C*^−1/3^. And varying *C* would be a practical way of experimentally changing the parameter *K* of the model. The variable *P*(*t*), represents the ensemble average of the projection of the dipole positions in the cartesian coordinates system: this is a spectroscopically measurable observable. Moreover, being related with the overall dipole moment of the solution, it can directly probe the emergence of a collective behaviour of the solvated molecules, collective behaviour which can only be driven by the presence of intermolecular interactions. A spectroscopic approach would thus entail a dichotomic, clear-cut, answer: if nothing would change in the absorption spectrum of the solutions at different concentrations, this would indicate that the solvated molecules do not interact at a (large) distance, to the contrary, concentration dependent spectral features would mean that the solvated molecules interact at a distance. In conclusion, the results reported in the present work outline a very promising experimental strategy - complementary to the diffusion-based one - to ascertain whether biomolecules can interact through long-range electrodynamic forces.

## Electronic supplementary material

Supplementary Information
