# Publisher Correction: Collective behavior of oscillating electric dipoles

**DOI:** 10.1038/s41598-020-60517-1

**Published:** 2020-04-03

**Authors:** Simona Olmi, Matteo Gori, Irene Donato, Marco Pettini

**Affiliations:** 1 Inria Sophia Antipolis Méditerranée Research Centre, MathNeuro Team, 2004 route des Lucioles-Boíte Postale 93, 06902 Sophia Antipolis, Cedex France; 2 Institut für Theoretische Physik, Technische Universität Berlin, Hardenbergstr. 36, 10623 Berlin, Germany; 3 CNR - Consiglio Nazionale delle Ricerche – Istituto dei Sistemi Complessi, 50019 Sesto Fiorentino, Italy; 4 Aix Marseille Univ, CNRS, CPT, Marseille, France; 5 CNRS Centre de Physique Théorique UMR7332, 13288 Marseille, France

Correction to: *Scientific Reports* 10.1038/s41598-018-33990-y, published online 24 October 2018

This Article contains errors. Reference 23 was inadvertently omitted and is given below as Reference 1.

In addition, there are errors in the reference citations.

In the ‘Numerical Results’ section,

“If we now investigate the role of the thermal noise strength, we obtain a stochastic resonance effect^18^: the signal at low frequency (≈0.28 ± 0.09) can be boosted by adding white noise to the signal, which contains a wide spectrum of frequencies.”

should read:

“If we now investigate the role of the thermal noise strength, we obtain a stochastic resonance effect^18,19^: the signal at low frequency (≈0.28 ± 0.09) can be boosted by adding white noise to the signal, which contains a wide spectrum of frequencies.”

In the ‘Discussion’ section,

“In particular chaotic irregular behavior emerging from regular unit dynamics has been seen in^19^, while the emergence of quasiperiodic motion has been shown in systems of oscillators^20^, neuron models^21^ and rotators^22^.”

should read:

“In particular chaotic irregular behavior emerging from regular unit dynamics has been seen in^20^, while the emergence of quasiperiodic motion has been shown in systems of oscillators^21^, neuron models^22^ and rotators^1^.”

Finally, the Acknowledgements section in this Article is incomplete.

“The authors acknowledge the financial support of the Future and Emerging Technologies (FET) Program within the Seventh Framework Program (FP7) for Research of the European Commission, under the FET-Proactive TOPDRIM Grant No. FP7-ICT-318121. S.O. thanks Stefano Lepri for useful discussions and suggestions and she acknowledges the Deutsche Forschungsgemeinschaft via Project A1 in the framework of SFB 910.”

should read:

“The authors acknowledge the financial support of the Future and Emerging Technologies (FET) Program within the Seventh Framework Program (FP7) for Research of the European Commission, under the FET-Proactive TOPDRIM Grant No. FP7-ICT-318121. The project leading to this publication has received funding also from the Excellence Initiative of Aix-Marseille University - A*Midex, a French “Investissements d’Avenir” programme. S.O. thanks Stefano Lepri for useful discussions and suggestions and she acknowledges the Deutsche Forschungsgemeinschaft via Project A1 in the framework of SFB 910.”
