# Supplementary material for: Collective behavior of oscillating electric dipoles
Source: Sci Rep. 2018 Oct 24;8:15748. doi: 10.1038/s41598-018-33990-y (PMC6200794; doi:10.1038/s41598-018-33990-y)
Supplement: Supplementary file 1 — Supplementary Information [file 41598_2018_33990_MOESM1_ESM.pdf]

SUPPLEMENTARY INFORMATION:
Collective behavior of oscillating electric dipoles

Simona Olmi,[1,2,3] Matteo Gori,[4,5] Irene Donato,[5] and Marco Pettini[4,5]

[1] *Inria Sophia Antipolis Méditerranée Research Centre, MathNeuro Team, 2004 route des Lucioles-Boîte Postale 93 06902 Sophia Antipolis, Cedex, France*

[2] *Institut für Theoretische Physik, Technische Universität Berlin, Hardenbergstr. 36, 10623 Berlin, Germany*

[3] *CNR - Consiglio Nazionale delle Ricerche - Istituto dei Sistemi Complessi, 50019, Sesto Fiorentino, Italy*

[4] *Aix Marseille Univ, CNRS, CPT, Marseille, France*

[5] *CNRS Centre de Physique Théorique UMR7332, 13288 Marseille, France*

(Dated: August 23, 2018)

PACS numbers: 87.10.Mn; 87.15.hg; 87.15.R-

I. DISCUSSION ABOUT CHARACTERISTIC TIME SCALES ON THE SYSTEM

The characteristic frequency for giant dipole oscillations has been conjectured to lie in a range between 0.1 − 10 THz so that the characteristic time for the oscillations is $\tau_{osc} \sim \omega_0^{-1} \simeq 10^{-13} - 10^{-11}s$. Recent experiments seem to provide a first evidence of the existence of collective biomolecule oscillations in this range of frequency in out-of-thermal equilibrium conditions. The characteristic time scale associated with translational diffusion of biomolecules can be estimated by

$$\tau_{trs} \approx \frac{\delta R^2}{6D_{trs}} \tag{1}$$

where δR is the tolerance in defining center of mass position of two biomolecules and D_{trs} is the self-diffusion coefficient of a biomolecule. We are interested in studing collective phenomena emerging due to long-range interactions in "diluted" system, meaning that the average intermolecular distance $\langle R \rangle \approx 10^{-7}$m is much larger then the characteristic molecular linear dimension scale $\lambda_{bio} \gtrsim 10^{-9}$m of biomolecules: this allows to consider $\delta R \approx \lambda_{bio}$. Using Einstein's formula for Brownian self-diffusion coefficient $D_{trs} = k_B T/(6\pi\eta_W \lambda_{bio})$ in eq. (1) we obtain

$$\tau_{trs} \simeq \frac{6\pi\lambda_{bio}^3}{k_B T} \gtrsim 3 \times 10^{-9}s \approx 10^2 \tau_{osc}. \tag{2}$$

This makes plausible the hypothesis that the center of mass of each biomolecule can be considered as a parameter and not a dynamical variable. Analogously, the characteristic time for biomolecules rotational diffusion has been estimated using

$$\tau_{rot} \approx D_{rot}^{-1} = \left(\frac{k_{Bol}T}{8\pi\eta_W \lambda_{bio}^3} \right)^{-1} \gtrsim 5 \times 10^{-9}s \approx 10^2 \tau_{osc}\,. \tag{3}$$

It follows that also diffusive rotation can be neglected on time scales characteristics for giant dipole oscillations and the orientation of dipoles can be assumed to be initially fixed.

II. LEVEL OF SYNCHRONIZATION IN THE SYSTEM

The collective evolution of the population and in particular the level of coherence is usually characterized in terms of the macroscopic field

$$\rho(t) = r_1(t)e^{i\Phi(t)} = \frac{1}{N}\sum_{j=1}^{N} e^{i\theta_j(t)}, \tag{4}$$

where the modulus r_1 is an order parameter for the synchronization transition being one ($\mathcal{O}(N^{-1/2})$) for synchronous (asynchronous) states, while Φ is the phase of the macroscopic indicator [1]. However, in our case, the molecules are pivoted to the center of mass and cannot rotate: the effective degree of freedom of these objects consists in an elongation/shrinkage along the direction identified by the mutual distance between the two centers of charges. Therefore it is not possible to describe the movement of the dipole in terms of an oscillator rotating along the unit-circle via the identification of a time-dependent phase. The solution that we have adopted is to calculate the phase of the single molecule by using the inversion formulas

$$\sin\theta_i = \frac{x_i - x_{0i}}{\sqrt{v_i^2 + (x_i - x_{0i})^2}}, \qquad \cos\theta_i = \frac{v_i}{\sqrt{v_i^2 + (x_i - x_{0i})^2}} \tag{5}$$

to associate a phase $\theta_i \in [-\pi, \pi]$ according to

$$\theta_i = \begin{cases} \arcsin(\sin\theta_i) & \text{if } \cos\theta_i \geq 0 \\ \pi - \arcsin(\sin\theta_i) & \text{if } \sin\theta_i > 0 \wedge \cos\theta_i < 0 \\ -\pi - \arcsin(\sin\theta_i) & \text{if } \sin\theta_i < 0 \wedge \cos\theta_i < 0. \end{cases} \tag{6}$$

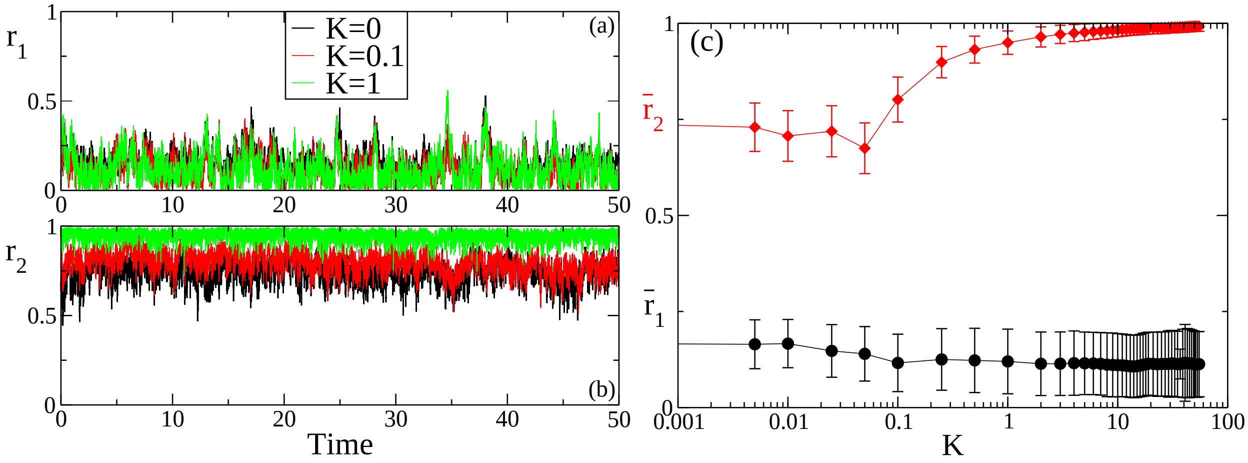

Figure 1: Synchronization properties of the system. Order parameters r_1 (a), r_2 (b) as a function of time for different coupling constants. Panel (c): Time-averaged order parameters as a function of the coupling constant K. The parameters values used for these simulations are: $\Omega_i = 0.01$, $x_{i0} = 5$, $\Omega_{frict,i} = 0.97$ (for every $i = 1, \ldots, N$), $\Omega_{pul} = 0.1$, $\mathcal{A}_{NE} = 0.011$, $N = 50$.

In the following we will present the analysis done for two sets of parameters, according to what we have already shown in Sec. III (Main Text). As a first set of parameters we consider

those corresponding to Figs. (1-4) in the main text. In this case the calculation of the order parameter r_1 does not lead to the identification of emergent (phase) synchronization in the system; in particular r_1 does not show any dependence on the coupling constant (see Fig. 1, panels (a) and (c)), as we would expect when the molecules are interacting with increasing strength. On the other hand if we calculate the order parameter usually employed to identify the emergence of 2-clusters ($r_2(t) = |\frac{1}{N}\sum_{j=1}^{N} e^{i2\theta_j(t)}|$), we clearly observe a transition to synchronization already for small coupling constants (see Fig. 1 (c)). This is confirmed if we calculate the distribution of positions and velocities of the molecules (see Fig. 2, panels (a)-(i)). In particular, if we look at the phase space (x, v) it emerges clearly that the system splits naturally in 2 clusters and the distance between the clusters increases if the single elements are coupled. The probability distribution functions of the positions reveal an increase of the elongation towards values that turn out to be not realistic from a bio-physical point of view if the coupling constant is too big ($K \geq 1$). Only the probability distribution profile of the velocities remains unchanged if the coupling constant and, therefore, the distance among the dipoles, is changed.

A better insight can be achieved if we looked at the average values and probability distribution functions of the phases. In absence of coupling the average phases are distributed in the interval $(-0.2, 0.2)$ (see Fig. 3 (a)). However, once the coupling is set, the profile of the time-averaged phases does not flatten sufficiently to show two distinct plateaus at two different phase values, thus allowing us two identify two phase-synchronized clusters (see Fig. 3 panels (d), (g)). Probably the approximation we have used to derive the phases as a function of positions and velocities (eq. 6), assuming still possible to invert the angle-action variables, is not sufficiently precise to capture the real dynamics of the system. Some more information can be deduced investigating the collective properties of the system, in particular by calculating the probability distribution function of the phases and visualizing the obtained results in terms of the corresponding effective free energy landscape, defined by $F(\theta) = -\ln(P(\theta))$, as plotted in Fig. 3 panels (c), (f), (i). The probability distributions of the phases present three peaks in correspondence of the angles $\theta = 0, -\pi, \pi$, see Fig. 3 panels (b), (e), (h), where the velocity of the oscillators is maxima; moreover, due to the formulation we have used, $+\pi$ and $-\pi$ are equivalent. This indicates that the system spends more time in these configurations, therefore we expect that the free energy landscape presents

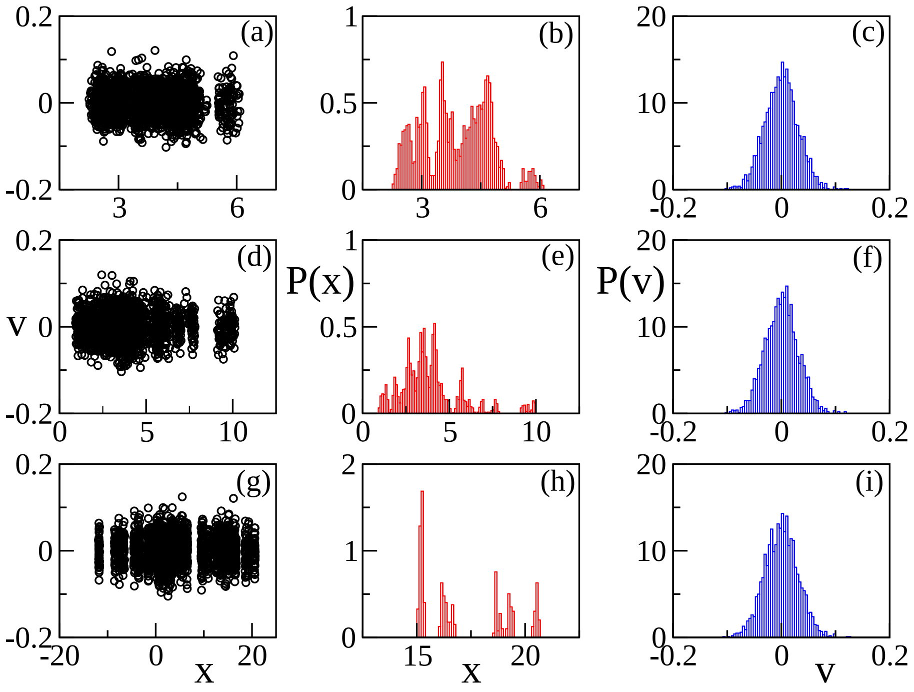

Figure 2: Panels (a), (d), (g): snapshots of the velocities of the single dipoles as a function of their positions for K=0 (a), K=0.1 (d), K=1 (g). Panels (b), (e), (h): probability distribution of the positions of the dipoles for different coupling constants. The panels refer to K=0 (b), K=0.1 (e), K=1 (h). Panels (c), (f), (i): probability distribution of the velocities of the dipoles for different coupling constants. The panels refer to K=0 (c), K=0.1 (f), K=1 (i). The probability distribution functions are obtained by measuring the corresponding variables at regular time intervals δT during a long simulation, after discarding an initial transient time. Parameters as in Fig. 1.

two minima, as the system preferably arranges itself along two specific angles. Indeed the free energy landscape $F(\theta)$ presents two peaks at $0, \pm\pi$, thus suggesting that these angles are more likely than the others and justifing the splitting in two clusters shown in Fig. 1(c), where the time-averaged behavior of r_2 is reported for increasing coupling strength.

Finally, a second set of parameters is investigated, corresponding to the Figs. (5, 6) shown in Sec. III (Main Text). Also in this second case the calculation of the order parameter r_1 does not lead to the identification of emergent (phase) synchronization in the system. In addition to this, the emergence of a collective behavior is not identifiable in a straightforward manner neither looking at the order parameter usually employed to identify the emergence

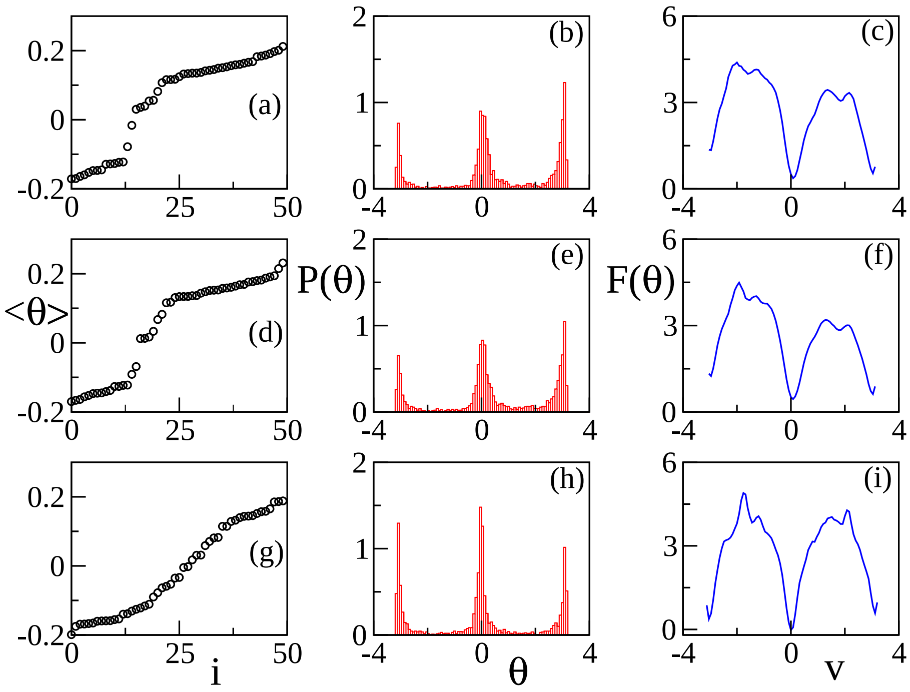

Figure 3: Panels (a),(d), (g): time-averaged phases as a function of the node index for K=0 (a), K=0.1 (d), K=1 (g). Panels (b), (e), (h): probability distribution of the phases of the dipoles for different coupling constants. The panels refer to K=0 (b), K=0.1 (e), K=1 (h). Panels (c), (f), (i): Effective free energy landscape $F(\theta) = -\ln(P(\theta))$ as obtained from the PDF of the phase $P(\theta)$. The panels refer to K=0 (c), K=0.1 (f), K=1 (i). The probability distribution functions are obtained by measuring the corresponding variables at regular time intervals δT during a long simulation, after discarding an initial transient time. Parameters as in Fig. 1.

of 2-clusters ($r_2(t) = |\frac{1}{N}\sum_{j=1}^{N} e^{i2\theta_j(t)}|$), nor at the distribution of positions and velocities of the molecules (see Fig. 4, panels (b)-(m)). Looking at the phase space (x, v) it does not emerge a clear separation in synchronized clusters among the dipoles and also the probability distributions of positions and velocities are simply Boltzmann-distributed, as we expect from a set of indepent oscillators subjected to a single asymmetric well potential in absence of coupling. For this second set of parameters the level of noise is much stronger, therefore a bigger coupling range is investigated. Only for very strong coupling (K=50) we can observe the emergence of a secondary small cluster in the phase space (x, v) (see Fig. 4(i)) that leads to a modification of the probability distribution of the positions, that is no more

simply Boltzmann-distributed, and to an increasing of the average value of $r_2(t)$ (see Fig. 4 panels (l) and (b) respectively).

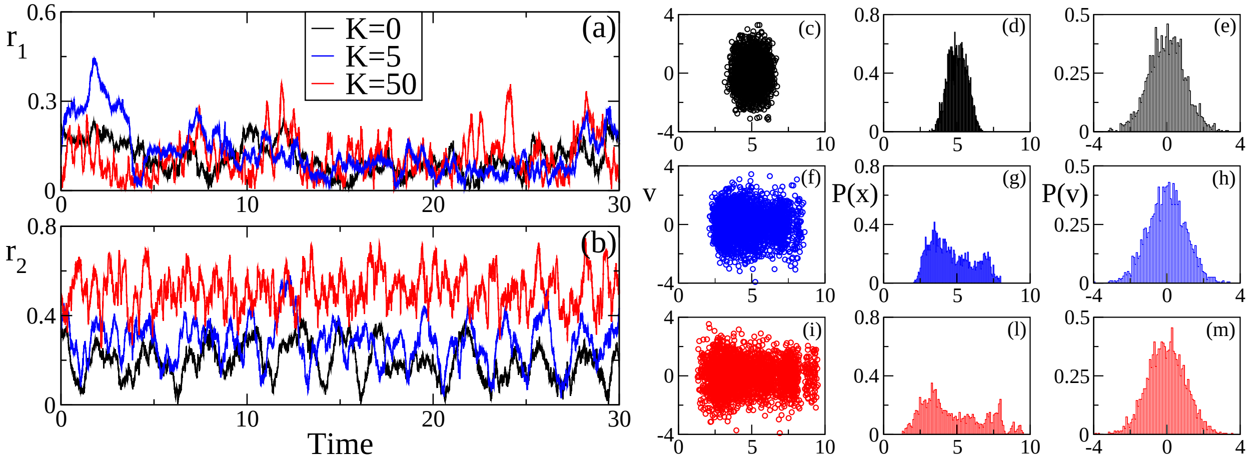

Figure 4: Synchronization properties of the system. Order parameters r_1 (a), r_2 (b) as a function of time for different coupling constants. Panels (c),(f), (i): snapshots of the velocities of the single dipoles as a function of their positions for K=0 (c), K=5 (f), K=50 (i). Panels (d), (g), (l): probability distribution of the positions of the dipoles for different coupling constants. The panels refer to K=0 (d), K=5 (g), K=50 (l). Panels (e), (h), (m): probability distribution of the velocities of the dipoles for different coupling constants. The panels refer to K=0 (e), K=5 (h), K=50 (m). The parameters values used for these simulations are: $\Omega_i = 1$, $x_{i0} = 5$, $\Omega_{frict,i} = 0.105$ (for every $i = 1, \ldots, N$), $\Omega_{pul} = 0.1$, $\mathcal{A}_{NE} = 1.4$, $N = 50$.

In conclusion the features of synchronization are not clearly identifiable in this system: for small noise levels (that correspond to settle the temperature of the system at T=300K) clustering effects are visible, enhanced by the proximity in space and confirmed by the shape of the effective free energy landscape; for higher noise values, phase synchronization cannot be achieved and it is even not possible to identify some form of cluster synchronization. In addition to this, the time behavior of the Kuramoto order parameter discards the possibility of having a synchronized state at the macroscopic level in both cases, since this would correspond to a constant behavior in time of r_1. On the other hand, the irregular behavior in time of the order parameter r_1 suggests a quasi-periodic or a chaotic behavior of the system at a macroscopic level. However, we have not calculated the maximum Lyapunov

exponent to discriminate among these two possibilities.

---

[1] Winfree, A. *The Geometry of Biological Time* (Springer, New York, 1980).
